# The case for an HIV cure and how to get there

**DOI:** 10.1016/S2352-3018(20)30232-0

**Published:** 2020-11-30

**Authors:** Mark Dybul, Timothy Attoye, Solange Baptiste, Peter Cherutich, François Dabis, Steven G Deeks, Carl Dieffenbach, Brian Doehle, Maureen M Goodenow, Adam Jiang, Dominic Kemps, Sharon R Lewin, Murray M Lumpkin, Lauren Mathae, Joseph M McCune, Thumbi Ndung'u, Moses Nsubuga, Holly L Peay, John Pottage, Mitchell Warren, Izukanji Sikazwe, Sunnylands 2019 Working Group

**Affiliations:** a Center for Global Health Practice and Impact, Georgetown University, Washington, DC, USA; b Global Health Division, The Bill & Melinda Gates Foundation, Seattle, WA, USA; c International Treatment Preparedness Coalition, Johannesburg, South Africa; d Kenya Ministry of Health, Nairobi, Kenya; e Agence Nationale de Recherches sur le SIDA et les Hepatites Virales, Paris, France; f University of California, San Francisco, California, USA; g Division of AIDS, National Institute of Allergy and Infectious Diseases, National Institutes of Health, Department of Health and Human Services, Rockville, MD, USA; h Office of AIDS Research, National Institutes of Health, Department of Health and Human Services, Rockville, MD, USA; i McKinsey & Company Secondee at The Bill & Melinda Gates Foundation, Seattle, WA, USA; j Sommartel, London, UK; k Peter Doherty Institute for Infection and Immunity, University of Melbourne and Royal Melbourne Hospital, Melbourne, Australia; l Department of Infectious Diseases, Alfred Hospital and Monash University, Melbourne, Australia; m Africa Health Research Institute, Durban, South Africa; n HIV Pathogenesis Programme, Doris Duke Medical Research Institute, University of KwaZulu-Natal, Durban South Africa; o Max Planck Institute for Infection Biology, Berlin, Germany; p University College London, London, UK; q Joint Adherent Brothers & Sisters Against AIDS, Kampala, Uganda; r RTI International, Research Triangle Park, NC, USA; s Viiv Healthcare, Brentford, UK; t AIDS Vaccine Advocacy Coalition, New York, NY, USA; u Centre for Infectious Disease Research in Zambia, Lusaka, Zambia

## Abstract

In light of the increasing global burden of new HIV infections, growing financial requirements, and shifting funding landscape, the global health community must accelerate the development and delivery of an HIV cure to complement existing prevention modalities. An effective curative intervention could prevent new infections, overcome the limitations of antiretroviral treatment, combat stigma and discrimination, and provide a sustainable financial solution for pandemic control. We propose steps to plan for an HIV cure now, including defining a target product profile and establishing the HIV Cure Africa Acceleration Partnership (HCAAP), a multidisciplinary public-private partnership that will catalyse and promote HIV cure research through diverse stakeholder engagement. HCAAP will convene stakeholders, including people living with HIV, at an early stage to accelerate the design, social acceptability, and rapid adoption of HIV-cure products.

## Introduction

Globally, approximately 38 million people live with HIV, and 1·7 million people are newly infected yearly.^1^, ^2^ Although new infections have substantially decreased since the peak of 2·9 million people in 1997, prevalence has steadily increased because of the successful scale-up of antiretroviral therapy (ART), increasing the lifespan of people with HIV.^3^ At current growth rates, over 42 million individuals will live with HIV by 2030.^1^, ^2^

HIV disease burden disproportionately affects lower-income and middle-income countries (LMICs). This is evident in sub-Saharan Africa, a region that accounts for less than 15% of the global population but 68% of people living with HIV or AIDS and 57% of new infections.^1^, ^4^ Although AIDS deaths have declined 46% since 2010, in southern and eastern Africa,^3^ sub-Saharan Africa is at an inflection point due to its burgeoning youth population, among the most vulnerable populations for HIV infection. These changing demographics could cause a resurgence in new infections.^5^

**Figure 1:**
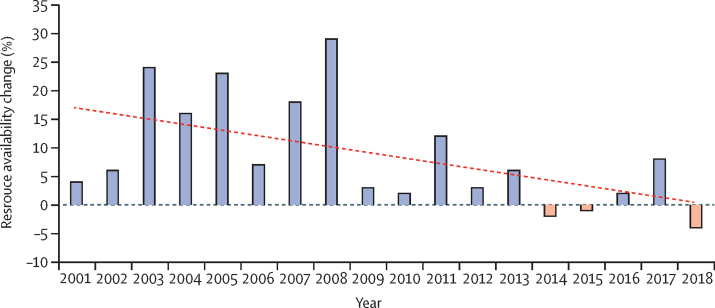
Year-on-year change in resource availability for HIV in low-income and middle-income countries Growth in total HIV resource availability for low-income and middle-income countries has declined in the past decade, with the largest year-on-year percentage decline since 2000 occurring in 2018.^1^ Dashed line is the linear regression best-fit line.

**Figure 2:**
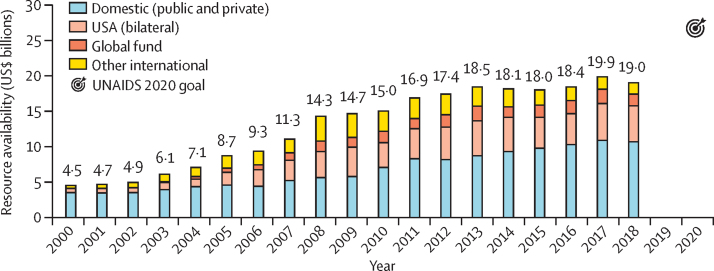
Total HIV resource availability for low-income and middle-income countries Total HIV resource availability for LMICs has stagnated in recent years and increasingly shifts towards domestic funding.^1^

Even as the total number of people living with HIV increases in LMICs, funding for HIV/AIDS programmes in these countries has not increased in recent years. Between 2000 and 2010, global funding to combat HIV/AIDS in LMICs grew from USD$4·5 billion to $15 billion, a 12·8% compound annual growth rate. Since 2010, growth has slowed to 2·9% compound annual growth rate; in the past 5 years, this has declined to 0·6% compound annual growth rate, barely reaching $19 billion in 2018 (figure 1). Although domestic funding has increased and now accounts for more than half of HIV resources in LMICs (figure 2), the growth is insufficient to achieve the UNAIDS 95-95-95 goals.^1^

Because of this landscape, the long-term sustainability of existing HIV and AIDS programmes is uncertain. Economic shocks (such as a recession) can further decrease resilience of the HIV response, highlighted by the economic instability caused by the 2019 novel coronavirus disease (COVID-19) pandemic. Not only has COVID-19 profoundly altered funding and advocacy for HIV, but health systems and human resources have also been stretched thin, and scientists researching HIV cures have shifted focus to promising therapies for the virus. The worst-case scenario could be that the gains of HIV control could be reversed, precipitating a loss of confidence, funding reductions, and increased incidence and mortality.

Here, we discuss how a therapeutic cure for HIV could address persistent unmet needs associated with current treatment and prevention strategies. We define an HIV cure as an intervention that leads to sustained HIV remission in an individual, suppressing HIV viraemia, minimising transmission, preventing re-infection, and maintaining indefinite viral control in the absence of ART.

Some argue that preparing for a cure now will further silo HIV services and divert scarce funding from other disease areas, or that it is too early to anticipate a cure because of the existing science. Conversely, we believe timely, successful roll out of any HIV cure product requires immediate coordinated action to avoid common implementation delays and infections or deaths that could be prevented with a cure. An HIV cure can prove highly cost-effective by mitigating long-term health and economic consequences of HIV, and eventually replace daily and long-acting treatment modalities; these cost savings could free up health resources for the treatment of other diseases. Moreover, an HIV cure could fortify cross-cutting services, infrastructure, information systems, and human resources within HIV programmes and across the wider health system, particularly if all relevant actors are engaged from the start. We propose a public sector and private sector partnership to influence the design and accelerate the development of an HIV cure. Five factors drive the need for an HIV cure: improving ART access and adherence; enhancing quality of life of people living with HIV; preventing new infections; combating stigma and discrimination; and ensuring financial and programmatic sustainability and scalability.

## Case for a cure

### Access and adherence

ART remains, at the individual level, one of the most effective tools to fight HIV and has shown global success in prolonging life and reducing risk of transmission. Yet ART is limited by several factors that can inhibit long-term retention and drive loss to follow-up.^6^, ^7^ Despite increased access to generics and free services, people living with HIV encounter barriers in accessing ART and related services and adhering to lifelong ART regimens (eg, treatment fatigue, pill burden, side-effects, job and food insecurity, stigmatisation, and health service dissatisfaction).^8^, ^9^, ^10^ Many LMICs face compounding structural barriers and service delivery inefficiencies such as long clinic and appointment wait times, long distances to treatment centres, and scarce supplies of quality-assured antiretrovirals.^8^, ^11^ Underserved areas and populations in high-income countries also experience social and structural obstacles to accessing otherwise widely available services, shown by the HIV epidemic in the USA and former Soviet Union countries.^12^, ^13^, ^14^, ^15^

Concerns regarding long-term toxicities of ART continue to emerge, such as the potential association between the integrase inhibitor class (the cornerstone of modern treatment) and obesity and neuropsychiatric disorders.^16^, ^17^, ^18^, ^19^, ^20^ Persistent immune activation and inflammation, even among virally suppressed individuals, are predictive of cardiovascular complications, cancers, osteoporosis, renal disease, neurocognitive disorders, and depression.^21^ Drug resistance can arise from poor ART adherence and suboptimal viral suppression rates, driven by weak health systems and treatment interruptions.^22^ Although some models suggest life expectancy on optimal ART approaches normal, ART initiation late in disease course and suboptimal adherence are still common.^23^, ^24^, ^25^ Even when individuals can freely access and remain durably suppressed on ART, life expectancy in many HIV-positive populations in high-income countries is nearly 10 years less than for age-matched, uninfected individuals.^26^, ^27^ With additional data, risk perception, and adherence might worsen.

### Quality of life

People living with HIV experience an overall lower quality of life. Individuals might cope with physical manifestations of HIV-related and treatment-related symptoms for extended periods, including fatigue, weight gain, pain, discomfort, and restricted mobility; even when on stable, long-term ART, individuals can experience comorbidities.^28^, ^29^, ^30^ People living with HIV also confront social and psychological challenges, such as the prospect of financial losses, depression, substance abuse, physical abuse, poor access to quality social support systems, and discrimination.^31^

### Prevention

Combination prevention packages frequently promote traditional interventions to modify sexual behaviours alongside biomedical interventions, including condoms, ART, voluntary medical male circumcision, and newer modalities such as pre-exposure prophylaxis (PrEP) and post-exposure prophylaxis (PEP). Although these have substantially improved pandemic control, social, economic, and psychological factors limit consistent use of each intervention. Moreover, no single intervention provides complete protection uniformly. Narrowly focused innovations and investments intended for demand creation or identifying efficiencies further complicate the prevention landscape.^32^

At the individual level, treatment as prevention can significantly reduce the risk of HIV transmission and will likely remain a keystone biomedical strategy to reduce incidence. At the population level, however, the effect of ART as a prevention modality has been underwhelming, due in part to delayed treatment initiation, poor adherence, and limited access to treatment.^33^, ^34^ More concerning, the ECHO study^35^ revealed high incidence rates among women in eastern and southern Africa with high ART coverage.

Recent modelling suggests an HIV cure, especially one that protects from re-infection, could reduce HIV incidence; the effect would be most profound in scenarios with poor access and adherence to ART, PrEP, and other interventions.^36^ Drawing from other sexually transmitted infections with high prevalence despite an available cure, the HIV cure agenda must actively engage people living with HIV to avoid perceptions of reduced risk or that discontinuing treatment will increase their chance of receiving a cure, factors that would mitigate the effect of a cure.

### Stigma and discrimination

Within health-care settings, HIV-related stigma and fear of discrimination discourage testing and prevention, delay treatment and enrollment in HIV care and services, and create confusion about transmission routes and risks.^37^ People living with HIV might also be denied health services, shown by data from the People Living with HIV Stigma Index.^38^, ^39^ Moreover, stigma and discrimination affect people living with HIV in education systems, justice systems, workplaces, families and communities, and emergency and humanitarian settings, as well as through self-stigma.^40^, ^41^, ^42^ Community, internalised, and anticipated stigma and discrimination frequently manifest as physical and emotional violence.^43^ Laws that discriminate against people living with HIV or key populations, such as criminalisation of HIV non-disclosure or same-sex partnerships, further institutionalise barriers to care.^39^, ^40^ Despite human rights approaches to confront stigma, vulnerable populations, who experience cross-sectional discrimination due to their perceived HIV status and identities, are disproportionately affected. Moreover, existing psychosocial services required to strengthen HIV care outcomes are chronically underfunded, where they exist at all.

### Sustainability

The UN General Assembly noted concerns about the sustainability of providing lifelong HIV treatment in the 2016 Political Declaration on HIV and AIDS: “If we accept the status quo unchanged, the epidemic will rebound in several developing countries, more people will acquire HIV and die from AIDS-related illness in 2030 than in 2015, and treatment costs will rise”.^44^ Such a rebound could select for isolates of HIV that are resistant to existing antiretroviral compounds and threaten the viability of new long-acting modalities.

Even a cure that is initially less safe, effective, or scalable than optimally delivered ART could contribute to pandemic control and lower the risk of a potential resurgence of the HIV/AIDS pandemic, aligning progress with global ambition.^36^ Long term, a cure that eliminates or durably suppresses HIV in an individual and prevents transmission could replace daily or long-acting treatment modalities and free global and domestic health resources for other priority issues, including emerging infectious diseases and the growing burden of non-communicable diseases in LMICs.^45^ Current fast-track goals to accelerate the HIV response in LMICs between 2015 and 2030, leveraging existing treatments, could provide substantial cost savings for treatment; the savings from an HIV cure could be far greater.^46^

## Case for preparing for a cure now

In the USA, the time frame from product discovery to regulatory approval extends to 10 years, and at least another 5 years to achieve widescale implementation and maximal market uptake.^47^ This process is often longer in LMICs. Acceleration of any effective HIV prevention or treatment intervention, particularly a potential cure, could rapidly reduce new infections, reduce stigmatisation, and increase financial and programmatic sustainability of the response.

Although a viable cure is not anticipated for at least a decade, an eventual cure could strengthen existing health systems and HIV programmes as it is developed and implemented at scale. Increased attention and resources for an HIV cure could concurrently drive financing, improve infrastructure, enhance health information systems and pharmacovigilance, and expand human resources in ways that benefit non-HIV services. Rigorous planning to integrate the HIV cure agenda into national health strategies will ensure that vertical programming to increase HIV cure uptake is harmonised with cross-cutting measures for broader health system strengthening.

## Developing target product profiles for HIV cure

Urgent development of a proof-of-concept HIV cure is needed, regardless of immediate global applicability. As with ART, once a concept is proven, market incentives and demand will likely galvanise more effective iterations, leading to a cure that could be very effective, safe, and scalable. However, early patient preference research and wide stakeholder engagement could inform earlier iterations and lead to globally applicable products from the outset. All affected parties, from communities to regulators and research and product developers, must be meaningfully engaged to define and to understand the problem, to propose solutions, to assess community reactions to proposed solutions, and to design metrics of success.

Despite existing global standards for such stakeholder engagement, including the Good Participatory Practice guidelines,^48^ community-level capacity building to develop treatment, cure, and research literacy will be required to ensure participation and leadership from HIV-endemic countries. Formal and informal channels can generate community input, including ongoing dialogue and community-based meetings and workshops led by people living with HIV, local and political social leaders, existing community-based organisations, community advisory boards, and activist groups that influence the ability of people living with HIV to access an HIV cure.^48^, ^49^, ^50^

*House on Fire: The Fight to Eradicate Smallpox*^51^ provides a good example of how engagement and research can generate greater effect. William Foege reveals that the shape of the smallpox vaccine delivery mechanism, a jet injector which resembled a gun, had a negative cultural meaning in some areas.^51^ Moreover, the jet injector was not widely accepted due to inconvenient transport, specialised training requirements, and high maintenance costs. Modifying the vaccination technique to the simpler bifurcated needle offered easier delivery, minimal training, simple sterilisation, reduced pain and trauma for patients, and ultimately increased vaccination rates.

Stringent regulatory approval of the antimalarial tafenoquine in the USA and Australia reiterates the value of stakeholder engagement during product development. Because of the risk of haemolysis from tafenoquine in individuals with G6PD deficiency, WHO and US Food and Drug Administration (FDA) could only recommend its use contingent on a companion, G6PD diagnostic.^52^ Although the FDA and Australia Therapeutic Goods Administration eventually approved tafenoquine in 2018, earlier collaboration with drug development stakeholders and companion diagnostics manufacturers likely would have minimised implementation delays.

Specific to HIV, scale-up of oral PrEP has been slow, despite receiving FDA approval in 2012, and providing near complete protection against HIV when taken daily.^53^, ^54^, ^55^ An estimated 350 000 individuals have ever used PrEP, with two-thirds of these in the USA, a far cry from the UN goal of 3 million users by 2020.^56^ Limited regulatory approval and guidance contribute to this gap; by 2019, only 46 countries approved a form of PrEP and 37 countries included PrEP in national guidelines. In some countries, access remains poor, available primarily for clinical research, demonstrations, or implementation projects.^57^ An absence of awareness and knowledge about PrEP, particularly among key populations, further limits global demand and uptake.^56^ In South Africa, for example, programmes to reach young women–a critical group for PrEP delivery–were developed only recently.^58^

## Generating insights for target product profiles

Development of target product profiles for future HIV interventions, including a potential cure, can facilitate early discussions with regulators, communities, policy makers, and procurers, and provide a platform to agree on criteria for success (eg, the data needed for regulatory, policy, and procurement agency acceptance; the product attributes needed for community and health-care system acceptance; and the product effect needed to justify the investment in time, money, and human resources). Importantly, target product profiles are living documents requiring regular updates based on user needs, technical advances, and changes in the therapeutic landscape. Target product profiles describe a range of variables, including mode of administration, target populations, efficacy, acceptable toxicity thresholds, target cost of goods sold, and storage and handling requirements. Each variable includes a minimum target to achieve the minimally acceptable level of global health effect, and the potential hazards to reaching that goal with mitigation plans; this serves as a “no” or ”go” decision point. Each variable also has an optimistic goal that identifies requirements to achieve broader, widespread use, and more rapid effect. These variables align all stakeholders by broadly defining the regimen or product.

Manufacturers in high-income countries have long used human-centred design and market research to design products;^59^ patient experience and preference data are similarly used in drug development and regulatory processes in Europe and the USA. Despite increased investments in human-centred design over the past decade, long-standing practices often remain unchanged, potential product limitations remain poorly understood, and the specific needs of populations for whom a product has the most potential value remain largely unmet. A recent push aims to act upon newly generated human-centred design insights (eg, in product research, implementation, and evaluation) to implement global health programmes more effectively.^60^

Early engagement of regulatory agencies, guideline authorities, funders, government, and non-government implementers, and civil society advocates is also required to strengthen the chain from target-product-profile research and development to reaching the end-user. These stakeholders are broadly aligned around building mutually beneficial relationships with affected individuals, shaping research together, and efficiently and effectively moving new quality HIV products and innovations to communities and individuals for widespread roll out.^48^ Their insights can inform subsequent product optimisations, reduce the timescale from product development to implementation, and improve community uptake.

Qualitative or quantitative social behavioural research approaches also generate implementation insights. These approaches can be done before, during, or after implementation to obtain data from affected communities (eg, people living with HIV, high risk populations, and clinicians) and better understand attitudes and behaviours, generate and test theories that inform the design of interventions and their goals, and build a stronger evidence base.^61^, ^62^

## Advancing target product profiles for HIV cure

The field will likely require multiple target product profiles based on modality and complexity of administration (eg, combination therapy, ex-vivo cellular or gene therapies, or in-vivo gene therapy), target population (eg, aviraemic on ART, viraemic on ART, or naive to ART), and delivery setting (eg, capacity for complex care). Regular plasma viral load testing will be essential after discontinuing ART to determine if someone is in remission or has achieved cure, with more frequent testing after initial interruption. A target product profile for more accurate, affordable, and accessible home-based or point-of-care diagnostic tests to monitor viral suppression can therefore also be required.

Public and private organisations engaged in product discovery and development will likely have restricted ability to disclose details about their product development process, and the characteristics of an “aspirational” or “ideal” target product profile may be beyond the boundaries of current scientific knowledge. Nonetheless, early stakeholder engagement in creating target product profiles could inform discovery and development of a more effective HIV cure by framing desired product attributes, showing product safety and efficacy, and identifying the services required for product delivery. Such a process could even catalyse imagination towards curative pathways and strategies not previously considered.

## Early sensitisation and planning at all levels for accelerated uptake

Efforts to coordinate HIV cure research engagement and advocacy have emerged, including formal partnerships between academia and industry. However, few use product development planning to link public and private research and development to international, national, and subnational awareness. Early engagement in both research and product development could facilitate policy making, funding, advocacy, and planning for implementation after a product comes to market.

Despite efforts to scale up effective HIV prevention interventions, uptake has been disappointing, partly because implementation and uptake strategies were not considered until late product development.^63^, ^64^ Examples of suboptimal implementation exist beyond HIV, including the malaria and Ebola vaccines. Learning from past efforts, the Global HIV Vaccine Enterprise echoes the call for early sensitisation, stakeholder mobilisation before regulatory approval, and investments in planning activities to accelerate the development of a preventive HIV vaccine.^65^ A similar framework is being prepared for the anticipated availability of long-acting antiretrovirals.^66^

Partially effective products will become available before highly effective products, as has been the case with HIV and malaria vaccine candidates. Precisely because a highly effective HIV cure is unlikely in the near term, now is the time to initiate systematic use of human-centred design, formative social-behavioural research, and early community-level and individual-level stakeholder engagement, which are increasingly used in Africa.^67^, ^68^ These are essential to understand and to manage expectations, ensure stakeholders are informed of the meaning and evolution of the HIV cure concept, and minimise obstacles to implementation as more effective interventions emerge.

## HIV Cure Africa Acceleration Partnership (HCAAP)

Recognising the challenges and opportunities of an HIV cure and clear need for a multidisciplinary approach, we propose HCAAP. This public-private partnership will catalyse HIV cure research and implementation by coordinating a forum in which different groups learn and work together, leveraging existing platforms and forums where possible. HCAAP will focus on LMICs in Africa, given their disproportionate disease burden, scarce resources, and unique demographic challenges.^5^ However, we anticipate the outputs and models developed by HCAAP will also have a substantial effect on HIV efforts in other resource-limited regions (eg, southeast and central Asia, eastern Europe, and some areas of resource-rich regions, including rural Europe and North America). To enable broad application and adaptation of its findings, HCAAP will freely publish outputs and learnings.

The proposed partnership builds on two types of engagement with impacted communities. First, HCAAP will strengthen long-term involvement of a multidisciplinary group, comprised of professionals in the natural and social sciences, payers, regulators, community leaders, and people living with HIV, across all activities to build models and programmes that are practical and acceptable from the stakeholder perspective, thus improving the likelihood of success. HCAAP leadership, comprised of stakeholder representatives, will maintain the partnership's ability to adapt to changing technology and stakeholders. Second, HCAAP will test specific models and programmes and obtain insights from impacted community members regarding target product profile-related topics such as acceptability, potential barriers, and facilitators to implementation. For both aims, success depends on African ownership. To mitigate against common power imbalances while ensuring diverse representation of community perspectives and concerns,^69^ HCAAP leadership and activities will be co-created with African stakeholders across the value chain.

HCAAP offers an adaptive process for engagement that convenes appropriate stakeholders to accelerate and influence the design of cure products for optimised implementation and to sensitise key individuals to promote rapid uptake of products, including those that are effective in smaller proportions of the community.

**Figure 3:**
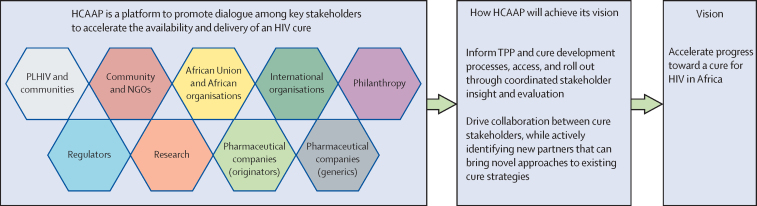
The HCAAP model HCAAP=HIV Cure Africa Acceleration Partnership. NGOs=non-governmental organisations. TPP=target product profile. PLHIV=people living with HIV.

To accomplish these goals, HCAAP will be established as an information sharing and strategic thinking public-private partnership, with the goal of providing academic and private-sector research and development, government health policy agencies, funders, regulators, and communities the tools and information required to advance timely access to products through their own respective processes (figure 3). Importantly, HCAAP intends to be a convening, not decision-making, body. Initially, HCAAP's primary activity will be to develop a definition of HIV cure and to test it in affected communities; to develop and adapt a target product profile for cure; and to elucidate, synthesise, and facilitate progress in the HIV cure field. Throughout this first phase, HCAAP will establish a viable and widely applicable process for early engagement to promote community ownership and rapid uptake of new HIV cure products. HCAAP members, particularly researchers from academia, industry, and external funders, will actively gather information and pipeline knowledge about the HIV cure space from their respective sectors. HCAAP could also do social-behavioural research to assess the feasibility and acceptability of target product profile components, or organise task forces to address specific challenges (eg, modelling, costing, regulation, scale-up, consumer insights, and communications); therefore, avoiding duplication across agencies.

In the medium-term, HCAAP could pivot to emerging candidate target product profiles and new HIV cure products. Recognising shortcomings in the current product development process, research and development companies have expressed interest in funding HCAAP to engage communities about their specific cure product concepts before clinical phases. HCAAP would ensure the larger community's voice is heard by all relevant stakeholders through a social-behavioural research approach, and stakeholders would determine how to best use these insights (eg, apply human-centred design, propose product modifications, carry out research studies to recommend manufacturer or deployment partners).

In the long term, the purpose and scope of HCAAP will evolve, as the International AIDS Vaccine Initiative evolved over its history. HCAAP could transition from a specialised and directive role focused on convenings and advocacy to an active collaborative role in research and product development (eg, identifying partners for current research funders). This evolution need not be predetermined; HCAAP must agilely adapt to changing science and stakeholder needs, assuring continuous community engagement in product design and maximising the potential for widespread effect of HIV curative interventions.

## Conclusion

An effective curative intervention, implemented as complementary to current and emerging treatment and prevention strategies, could catalyse pandemic control by treating people living with HIV who cannot access or adhere to ART, reducing barriers related to lifelong treatment, easing the global financial burden of long-term HIV treatment, and allowing for resource redirection. We believe convening people living with HIV and the broader HIV community in early development of a cure can accelerate education, acceptance, and adoption. Beyond HIV, this collaborative and multidisciplinary umbrella initiative could provide actionable best practices and the organisational backbone needed to anticipate challenges associated with product development and deployment for other diseases, including the urgent need for a COVID-19 vaccine. In anticipation of future public health crises, HCAAP could prompt efforts to extend the reach of scarce financial, advocacy, and workforce resources for HIV cure, draw on underutilised communities and stakeholders to lead the HIV response, and prioritise new workflows to sustain momentum. Now is the time to start.
